# Neuroprotective effect of omega 3‐oil against aluminium chloride induced oxidative stress in the cerebellum of adult wistar rat

**DOI:** 10.1002/alz.084833

**Published:** 2025-01-03

**Authors:** Cynthia Nyen Tangban

**Affiliations:** ^1^ University of Ibadan, Ibadan, Oyo Nigeria

## Abstract

**Background:**

The brain is a potential target for aluminium toxicity as it induces oxidative stress, strategies, rich in polyphenolic compound, containing flavonoid and possessing antioxidant property, found in natural plant products, to attenuate aluminium‐induced impairments could provide a potential therapeutic intervention and protection for aluminium neurotoxicity.

**Method:**

Forty adult rats weighing between 160 – 165g was used. The rats were divided into four groups (n = 10). Group (I) received distilled water group (II) received 100mg/kg body weight of aluminum chloride, group (III) received 200mg/kg of omega‐3 oil orally and group (IV) received 200mg/kg of omega‐3 oil and 100mg/kg of aluminum chloride in an hour interval for 28 days. The initial and final body weights as well as neurobehavioural studies were done on day 27 before sacrifice on day 29. The brain of the rats in each group was dissected out. Some cerebellar were preserved in phosphate buffered saline at 4°C and pH, 7.2 for oxidative stress and antioxidant markers, while other cerebellar were fixed in 10% formol‐saline for histological and immunohistochemical studies. Data analyzed using one way analysis of variance (ANOVA) at p<0.05.

**Result:**

The results showed normal activities in the control, omega‐3, aluminium+omega‐3 groups throughout the experimental periods while the group administered aluminium chloride showed general body weakness, decline in food and water intake, decrease body weight, alongside disturbance in the exploratory movement, anxiety, motor function and motor activity. Alcl3 induced oxidative stress by increasing lipid peroxidation and decreasing glutathione levels, glutathione peroxidase and catalase activities in the cerebellum compared with the control and other treated groups.histological observations showed distortion of the cytoarchitecture of the cerebellar cortex induced by aluminum which was evident by the severe loss of Purkinje cells and vacuolization of cells in the granular layer. Observations employing Glial Fibrillary Acidic Protein showed increased astrogliosis and apoptosis in the aluminum‐treated group compared with the other groups.

**Conclusion:**

Omega‐3 fatty oil reversed the neurological deficits induced by aluminium chloride. From the study, omega‐3 fatty acid decreased the rate at which aluminium chloride induced oxidative stress in the cerebellum of Wistar rats by increasing the antioxidant capacity of the brain.

